# Expression profile analysis to predict potential biomarkers for glaucoma: BMP1, DMD and GEM

**DOI:** 10.7717/peerj.9462

**Published:** 2020-09-03

**Authors:** Dao wei Zhang, Shenghai Zhang, Jihong Wu

**Affiliations:** 1 Eye Institute, Eye and ENT Hospital, College of Medicine, Fudan University, Shanghai, China; 2 Shanghai Key Laboratory of Visual Impairment and Restoration, Science and Technology Commission of Shanghai Municipality, Shanghai, China; 3 State Key Laboratory of Medical Neurobiology, Institutes of Brain Science and Collaborative Innovation Center for Brain Science, Shanghai, China; 4 Key Laboratory of Myopia, Ministry of Health, Shanghai, China

**Keywords:** Glaucoma, WGCNA, Gene biomarker, Prognosis, BMP, DMD, GEM

## Abstract

**Purpose:**

Glaucoma is the second commonest cause of blindness. We assessed the gene expression profile of astrocytes in the optic nerve head to identify possible prognostic biomarkers for glaucoma.

**Method:**

A total of 20 patient and nine normal control subject samples were derived from the GSE9944 (six normal samples and 13 patient samples) and GSE2378 (three normal samples and seven patient samples) datasets, screened by microarray-tested optic nerve head tissues, were obtained from the Gene Expression Omnibus (GEO) database. We used a weighted gene coexpression network analysis (WGCNA) to identify coexpressed gene modules. We also performed a functional enrichment analysis and least absolute shrinkage and selection operator (LASSO) regression analysis. Genes expression was represented by boxplots, functional geneset enrichment analyses (GSEA) were used to profile the expression patterns of all the key genes. Then the key genes were validated by the external dataset.

**Results:**

A total 8,606 genes and 19 human optic nerve head samples taken from glaucoma patients in the GSE9944 were compared with normal control samples to construct the co-expression gene modules. After selecting the most common clinical traits of glaucoma, their association with gene expression was established, which sorted two modules showing greatest correlations. One with the correlation coefficient is 0.56 (*P* = 0.01) and the other with the correlation coefficient is −0.56 (*P* = 0.01). Hub genes of these modules were identified using scatterplots of gene significance versus module membership. A functional enrichment analysis showed that the former module was mainly enriched in genes involved in cellular inflammation and injury, whereas the latter was mainly enriched in genes involved in tissue homeostasis and physiological processes. This suggests that genes in the green–yellow module may play critical roles in the onset and development of glaucoma. A LASSO regression analysis identified three hub genes: Recombinant Bone Morphogenetic Protein 1 gene (*BMP1*), Duchenne muscular dystrophy gene (*DMD*) and mitogens induced GTP-binding protein gene (*GEM*). The expression levels of the three genes in the glaucoma group were significantly lower than those in the normal group. GSEA further illuminated that *BMP1*,* DMD* and* GEM* participated in the occurrence and development of some important metabolic progresses. Using the GSE2378 dataset, we confirmed the high validity of the model, with an area under the receiver operator characteristic curve of 85%.

**Conclusion:**

We identified several key genes, including *BMP1*, *DMD* and *GEM,* that may be involved in the pathogenesis of glaucoma. Our results may help to determine the prognosis of glaucoma and/or to design gene- or molecule-targeted drugs.

## Introduction

Glaucoma is the leading cause of irreversible blindness worldwide (Jonas et al., 2017). Like tumors and cardiovascular disorders, it is strongly associated with the deterioration of quality of life, therefore it is an important medical problem that requires treatment (Quaranta et al., 2016). Gene expression aberrations have been widely recognized to participate in the pathological process of tumor (Flavahan, Gaskell & Bernstein, 2017). This abnormality is not limited to tumors, but has been linked to a variety of ophthalmopathy (Heavner & Pevny, 2012; Solinis et al., 2015), thus we assume that they also contribute to the development and progression of glaucoma. Systemic diseases, genetic factors, and environmental factors may also play critical roles in the pathogenesis of glaucoma. Long-term studies have repeatedly shown that elevated intraocular pressure (IOP), age, and genetics are the main risk factors for glaucoma (Doucette et al., 2015; McMonnies, 2017). However, the treatment of glaucoma is limited to drugs that lower IOP (Lusthaus & Goldberg, 2016) and surgical therapy (Fenwick et al., 2019), which may temporarily alleviate IOP.

Glaucoma is generally characterized by a reduction in the number of retinal ganglion cells, the thinning of the retinal nerve fiber layer, and the cupping of the optic disc. The optic nerve head is a key anatomical site that shows signs of early glaucomatous damage (Minckler, Bunt & Johanson, 1977). In addition to pathological changes of the retinal ganglion cells, the optic nerve head undergoes reactive astrocyte remodeling, particularly in response to stress or other pathological disorders. This can lead to a series of morphologic, gene expression, and functional changes (Sofroniew & Vinters, 2010). It has also been reported that astrocytes play a key role in the disease process, but there is limited evidence about the specific pathological events that occur in these cells.

In recent decades, advances in bioinformatics, high-throughput sequencing, and genetic association studies have greatly accelerated the discovery of genes and genomic regions involved in ophthalmic diseases, including glaucoma. These studies have demonstrated that glaucoma is a complex disease (MacGregor et al., 2018). Previous genome-wide association studies identified over 10 genes associated with primary open-angle glaucoma, including myocilin (*MYOC*), optineurin (*OPTN*) and WD repeat domain 36 (*WDR36*) (Liu & Allingham, 2017). Mutations in *MYOC* cause a cascade of abnormalities in the trabecular meshwork, including the intracellular retention of myocilin, reduced aqueous outflow, increased IOP, and glaucoma (Fini, 2017). Optineurin regulates many physiological processes, including membrane trafficking, protein secretion, cell division, autophagy, and host defense against pathogens (Kachaner et al., 2012). WDR36 is a nucleolar protein involved in the maturation of 18S rRNA and the mutation of *WDR36* can delay the formation of 18S rRNA and the apoptosis of human trabecular meshwork cells. A number of other genes are also involved in secondary glaucoma, including *SBF2*, *EPHA2*, *TRPM3* and *TMEM98* (Doucette et al., 2015).

In this study, we sought to identify novel biomarkers associated with the pathogenesis of glaucoma. To achieve this, we used a weighted gene coexpression network analysis (WGCNA) to construct a network of coexpressed genes, in order to describe the correlations among genes across multiple samples. Although WGCNA may not be as effective as other techniques in identifying modules with high functional relevance and biological significance, it is the most widely used for this purpose (Kakati et al., 2019). We used this correlation-based gene screening method to identify candidate biomarkers and/or therapeutic targets for glaucoma.

## Material and Methods

### Data source

We searched the Gene Expression Omnibus (GEO) database (http://www.ncbi.nlm.nih.gov/geo/) using the keyword “glaucoma”. The datasets were yielded according to the following criteria: (1) datasets contain samples from both normal and glaucoma patients along with necessary clinical characteristics such as gender and age; (2) datasets exhibit original expression profiles derived from microarray which already had been background corrected and standardized; (3) datasets have a relatively sufficient sample size for further analysis. Two datasets were selected with GSE9944 (Lukas et al., 2008) using as the training dataset and GSE2378 (Hernandez et al., 2002) as the validation dataset.

### WGCNA

WGCNA is a bioinformatics tool that we applied to construct the expression patterns of genes from multiple samples, generating clusters of genes with similar expression patterns, allowing the researcher to analyze the correlations between modules and specific traits or phenotypes (Langfelder & Horvath, 2008). To identify genes related to glaucoma, we assumed that the gene networks obeyed a scale-free distribution in the WGCNA. Pairwise Pearson’s correlation coefficients were used to evaluate the co-expression relationships among all the genes, and were converted into a scale-free network by transforming the correlation matrix to an adjacency matrix with a soft threshold value, which specified the range of changes in the detected data. The soft threshold value was selected as the standard of a scale-free distribution. Based on the adjacency matrix, genes with absolute high correlation were clustered into the same module (Feltrin et al., 2019). The dissimilarity of the topological matrix was then incorporated into an unsupervised hierarchical cluster with a dynamic tree-cutting algorithm. The branches of the clusters were defined as modules (Oros Klein et al., 2016) and each module represented a specific gene expression profile that was generalized by the main component eigengene (Zhang et al., 2017). The scatterplots of gene significance and module membership were painted to define hub genes. All the above processes of WGCNA were the realization by R program. Besides, the topological overlap of intramodules and adjacency modules was used for selecting the functional modules, and modules with low or no correlation to glaucoma (*P* value ≥ 0.01) were excluded.

### Functional enrichment analysis

A functional enrichment analysis was performed on the modules most relevant to the glaucoma traits using Metascape (http://metascape.org/), which utilized the well-adopted hypergeometric test and Benjamini-Hochberg *p*-value correction algorithm to identify all the genes. We identified all the statistically significantly enriched terms by calculating the cumulative hypergeometric *P* values and enrichment factors, which were then used to filter the data. The significant terms were then hierarchically clustered into a tree based on the statistical similarities (derived from the *κ* values) among the member genes. A *κ* value of 0.3 was used as the threshold to divide the tree into term clusters. A *P* value of <0.01 was used as the screening threshold for significant pathways (Zhou et al., 2019).

### LASSO regression analysis

We performed LASSO regression analysis with integration of the genes in the modules by using the glmnet R package (version 2.0.16, https://cran.r-project.org/web/packages/glmnet) (Friedman, Hastie & Tibshirani, 2010), as our purpose was to extract the precise expression quantity resorting dimensionality reduction algorithm. We regularized the LASSO regression coefficient to prevent over-fitting the results in order to screen the key genes.

### Gene set enrichment analysis

In order to compare difference in glaucoma group and normal group, we first applied the point graphs to represent genes expression levels in the GSE9944. Geneset enrichment analyses (GSEA) was divided into high and low groups according to the median value of gene expression, and then analyzed with GSEA enrichment software (version 4.0.2, http://software.broadinstitute.org/gsea/index.jsp) (Subramanian et al., 2005). The significant pathway was presented by a table whose screening condition was *P* value <0.05. GSEA was performed to identify the pathways associated with key genes (Ye et al., 2019) in the training dataset.

### External validation of key genes

The key genes identified with the training dataset were evaluated the accuracy of these genes with a logistic regression analysis using the pROC R package (version : 1.16.1, https://cran.r-project.org/web/packages/pROC) (Robin et al., 2011). Key genes were validated via the external dataset (GSE2378) with a receiver operating characteristic (ROC) curve analysis to determine the sensitivity and specificity of the analysis. The area under the curve (AUC) represents the likelihood that the eigengene can be considered as biomarker (Yu et al., 2018).

### Statistical analysis

The two.sided wilcox test was used to determine the statistical significance when comparing the glaucoma tissue with the normal tissue. Statistical analysis was carried out using R software, version 3.6.2.

## Results

### Sample traits and data

Two gene expression datasets of optic nerve head astrocytes (GSE9944 and GSE2378) obtained from donors with or without glaucoma were downloaded from GEO in October 2019, and the GPL version of two datasets with the largest disease samples size was selected for further analysis. The training dataset, GSE9944 dataset, comprised 63 samples, and 13 patient samples and 6 normal samples were finally selected for WGCNA to screen potential genes. The GSE2378 dataset comprised 15 samples in two GPLs, we chose the one with a larger sample size (*N* = 13). Among these 13 samples, however, there were three samples without clarification of disease or normal, so we finally kept 10 samples as the external validation dataset.

### Glaucoma-related WGCNA modules and genes

**Figure 1:**
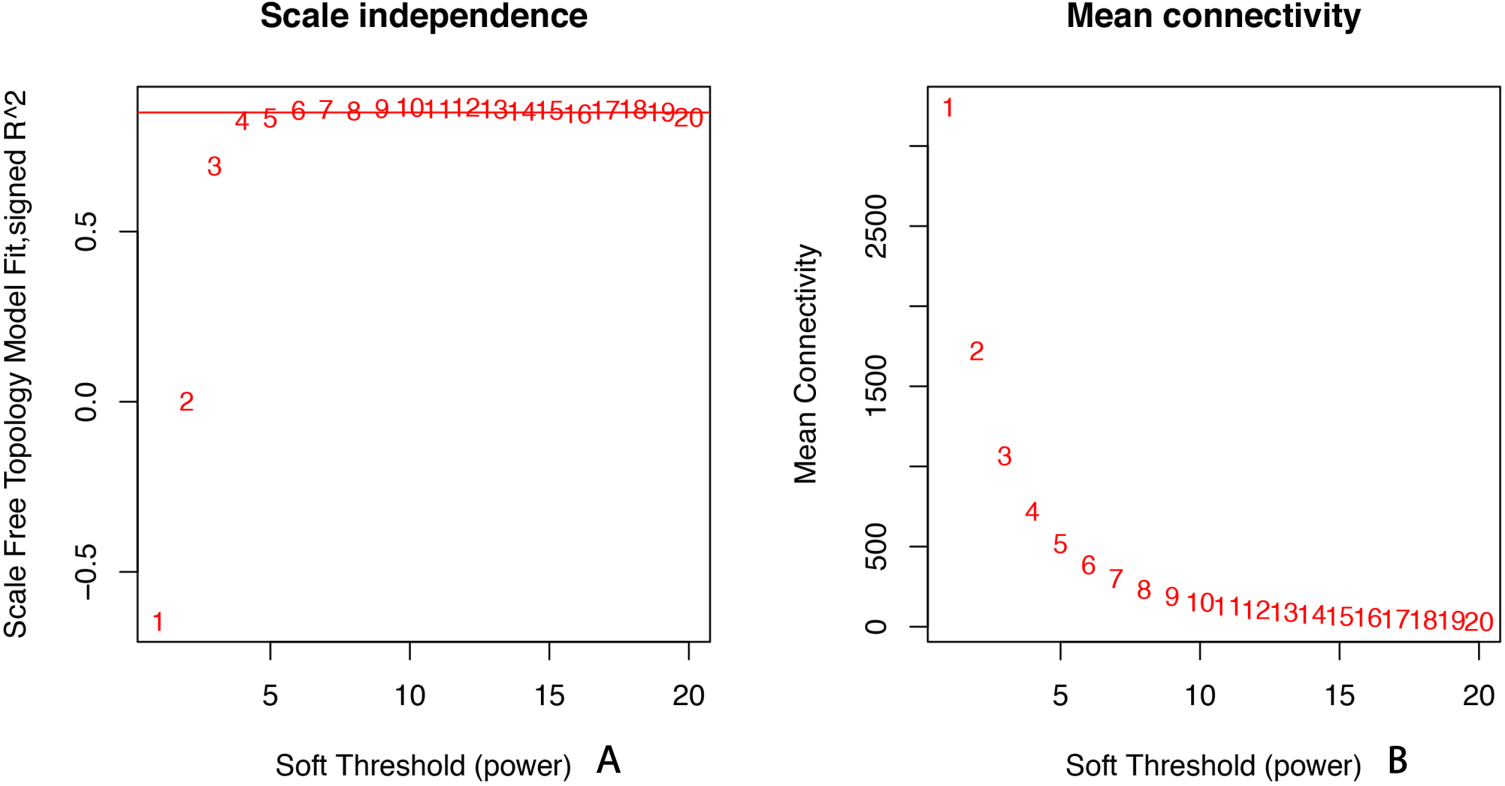
Selection of soft threshold powers. (A) Scale independence. Analysis of scale-free topology fit index for soft threshold powers (*β*). Red line indicates the correlation coefficient (0.85). (B) Mean connectivity of gene co-expression modules for different soft threshold powers.

**Figure 2:**
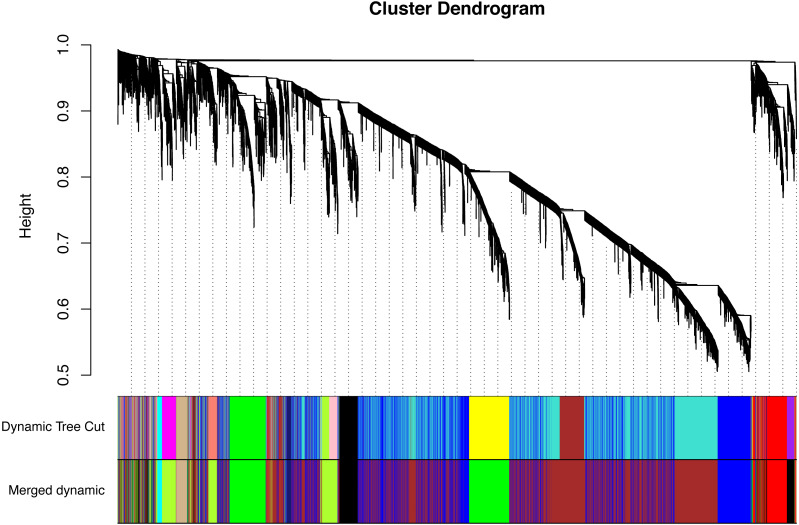
Identification of a co-expression module in glaucoma. The branches of the cluster dendrogram correspond to the 10 gene modules. Each piece of the leaves on the cluster dendrogram corresponds to a different gene module.

**Figure 3:**
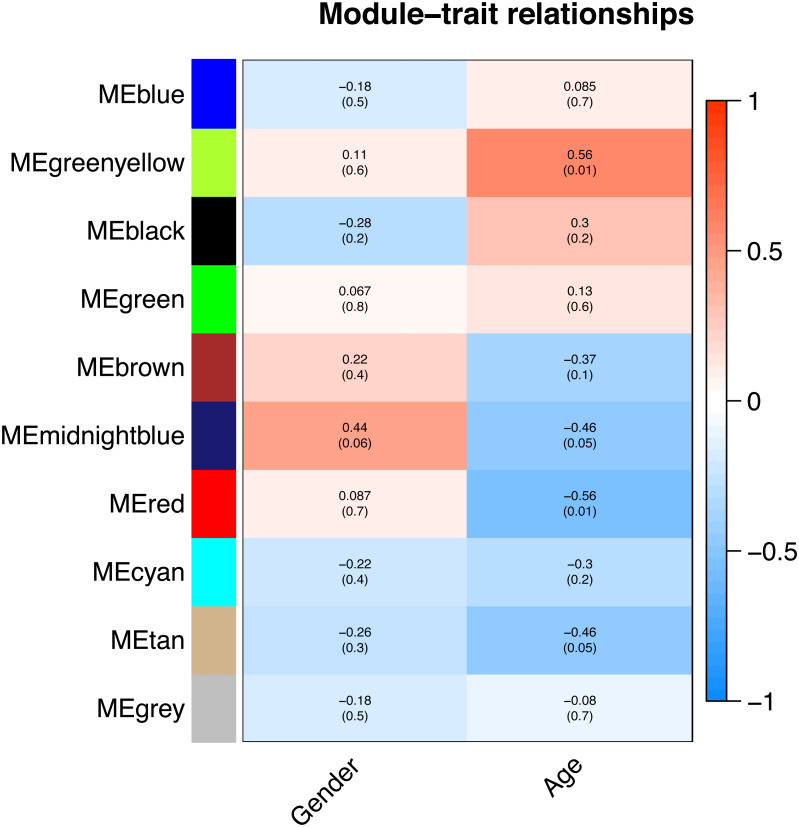
Correlation between the gene module and clinical traits. Gender and Age are included. The correlation coefficient in each cell represented the correlation between the gene module and the clinical traits. The corresponding *P*-value is also annotated. The green-yellow and the red were the most positively and negatively relevant modules to age, respectively.

All of the genes included in the GEO dataset were subjected to WGCNA. The top 25% of genes that showed the greatest variance were retained for subsequent WGCNA analysis, and ultimately 8,086 genes and 19 samples were included. For network analysis, the soft threshold power for matrix transformation was confirmed to be 6, the scale independence was 0.85, and the mean connectivity of the co-expression network was high to ensure a scale-free network (Fig. 1). We constructed the co-expression modules and identified 10 glaucoma-related modules, which were arbitrarily designated black (440 genes), blue (2,053 genes), brown (3,706 genes), cyan (65 genes), green (1,088 genes), green-yellow (674 genes), gray (5 genes), midnight-blue (59 genes), red (364 genes), and tan (125 genes) (Fig. 2). We selected the most common clinical traits (gender and age) of disease and then linked them to the gene expression modules based on the correlations between the eigengenes and the clinical traits of common expression pattern modules. As shown in Fig. 3, gender and age were included in the module–trait analyses. The green-yellow (*r* = 0.56, *P* = 0.01) and red modules (*r* =  − 0.56, *P* = 0.01) were strongly associated with age.

**Figure 4:**
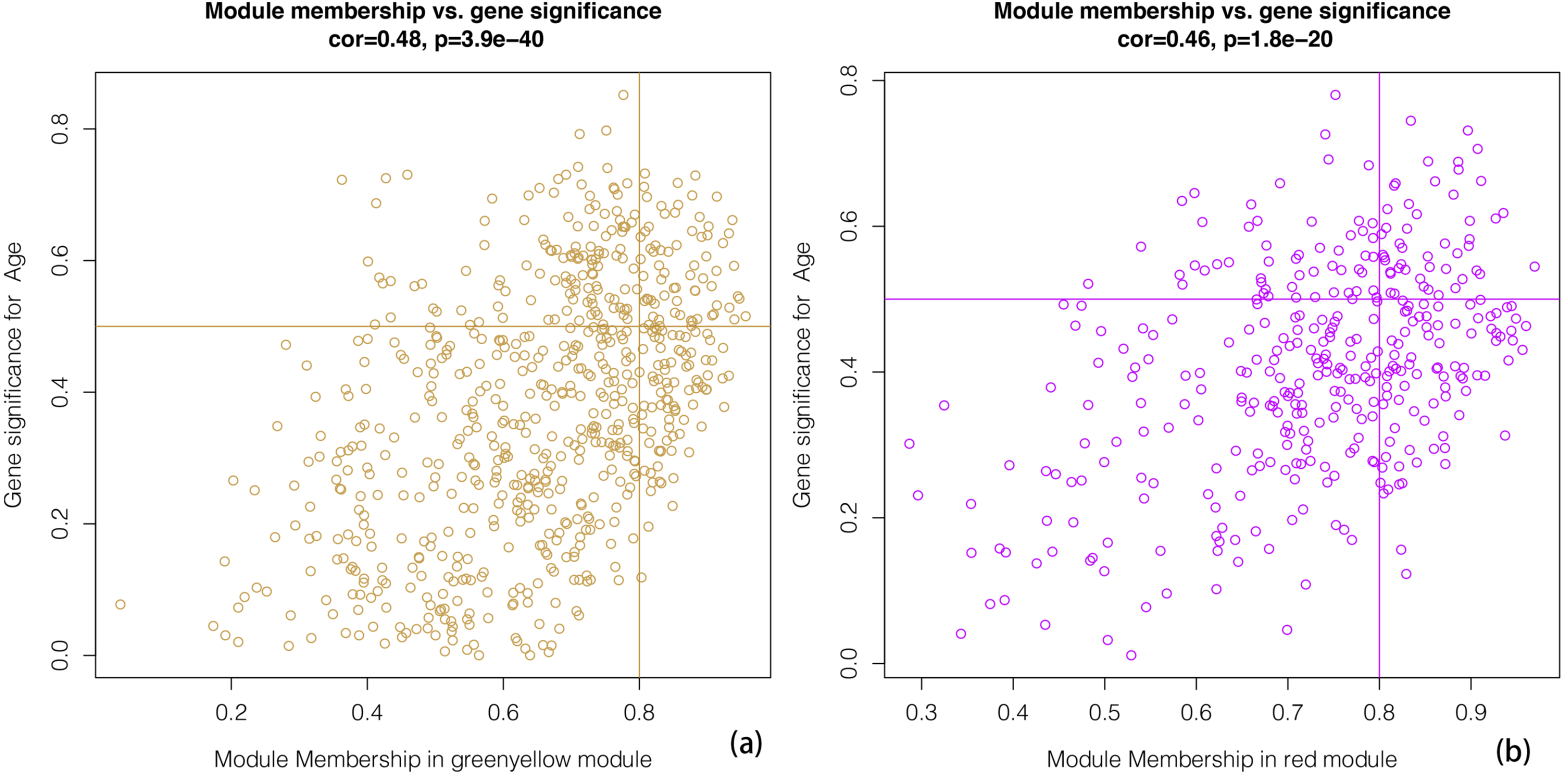
A scatter plot of Gene Significance (GS) vs. Module Membership (MM). A scatter plot of Gene Significance (GS) vs. Module Membership (MM) in the greenyellow (A). A scatter plot of Gene Significance (GS) vs. Module Membership (MM) in the red (B). We define cone genes as which absolute value of Gene Signicance is greater than 0.5 and the absolute value of Module Membership is greater than 0.8.

We constructed scatterplots of gene significance versus module membership for the green-yellow and red modules (Fig. 4). Genes with an absolute gene significance score of >0.5 and an absolute module membership score of >0.8 were regarded as the core genes in each module.

### Enrichment analysis

**Figure 5:**
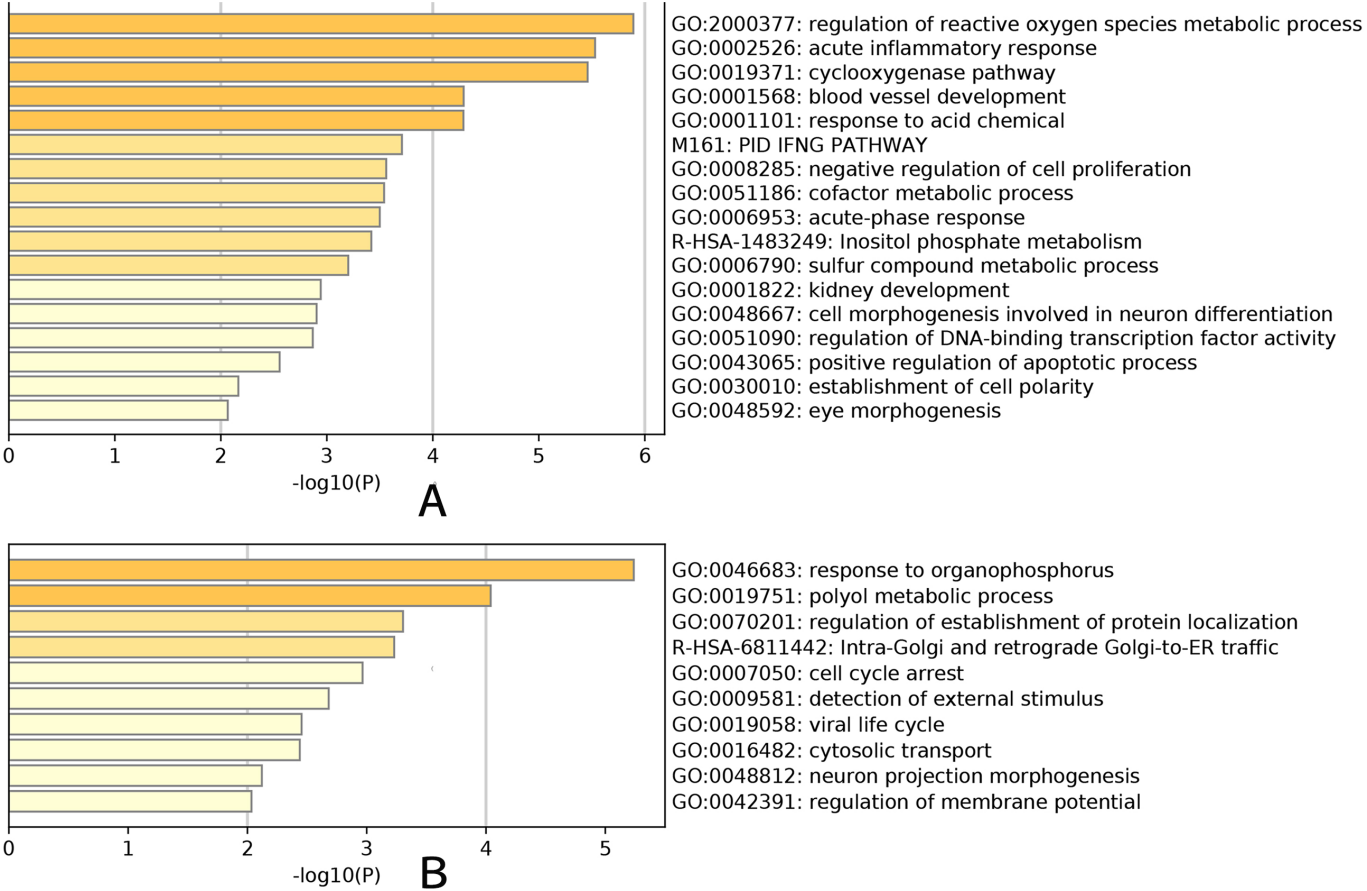
GO analysis. Enrichment analysis result for the yellow-green module (A). Enrichment analysis result for the red module (B). Yellow-green module was significantly enriched in development of glaucoma, especially in regulation of reactive oxygen species metabolic process (GO:2000377), acute inflammatory response (GO:0002526), cyclooxygenase pathway (GO:0019371), blood vessel development (GO:0001568) and response to acid chemical (GO:0001101). Genes in red module were significantly enriched in response to organophosphorus (GO:0046683) and polyol metabolic process (GO:0019751).

An enrichment analysis of the core genes based on Gene Ontology (GO) revealed that the green–yellow module was significantly enriched in genes involved in the development of glaucoma, particularly the regulation of reactive oxygen species metabolic processes (GO: 2000377), acute inflammatory responses (GO: 0002526), the cyclooxygenase pathway (GO: 0019371), blood vessel development (GO: 0001568), and the response to acidic chemicals (GO: 0001101) (Fig. 5A). The red module was significantly enriched in genes involved in the response to organophosphorus (GO: 0046683) and polyol metabolic processes (GO: 0019751) (Fig. 5B). These results demonstrated the marked differences in the genes present in each module, and that the green-yellow module was predominantly enriched in genes involved in cellular inflammation and injury. Based on gene enrichment, we speculated that the green–yellow module was the most important module in age-related glaucoma. By contrast, the red module was mainly enriched in genes involved in tissue physiological processes and cell cycles, which suggested that the encoded proteins may act in the regulation of pathological processes in these cells.

### LASSO regression analysis

**Figure 6:**
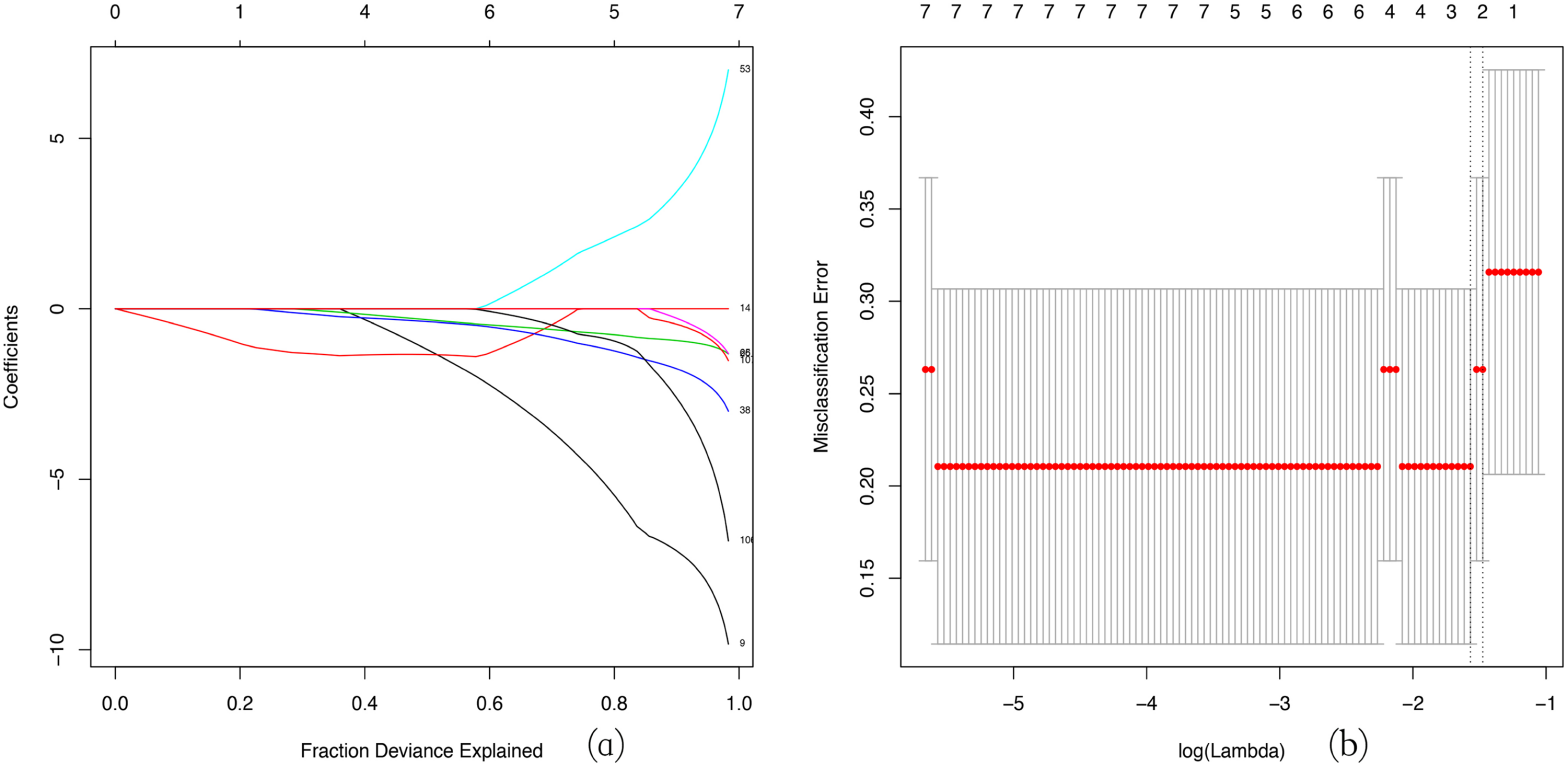
LASSO regression analysis. LASSO coefficient profiles of the module genes (A). Selection of the tuning parameter (*λ*) in the LASSO model through cross-validation procedure was plotted as a function of log (*λ*) (B). The y-axis represents Misclassification Error, and the lower x-axis represents the log (*λ*). Numbers along the upper x-axis represent the average number of predictors. Red dots indicate average deviance values for each model with a given *λ*, where the model provides its best fit to data.

LASSO regression is widely used in tumor analysis (He et al., 2019; Zhao et al., 2015), but is rarely applied in other diseases, especially to use WGCNA and LASSO regression analysis to screen hub gene together, which is an innovative aspect of our research. We used a LASSO regression analysis to identify the key genes in the green-yellow and red modules. Then we screened three key genes: *BMP1*, *DMD* and *GEM* in green–yellow module as a consequence (Fig. 6).

### GESA

**Figure 7:**
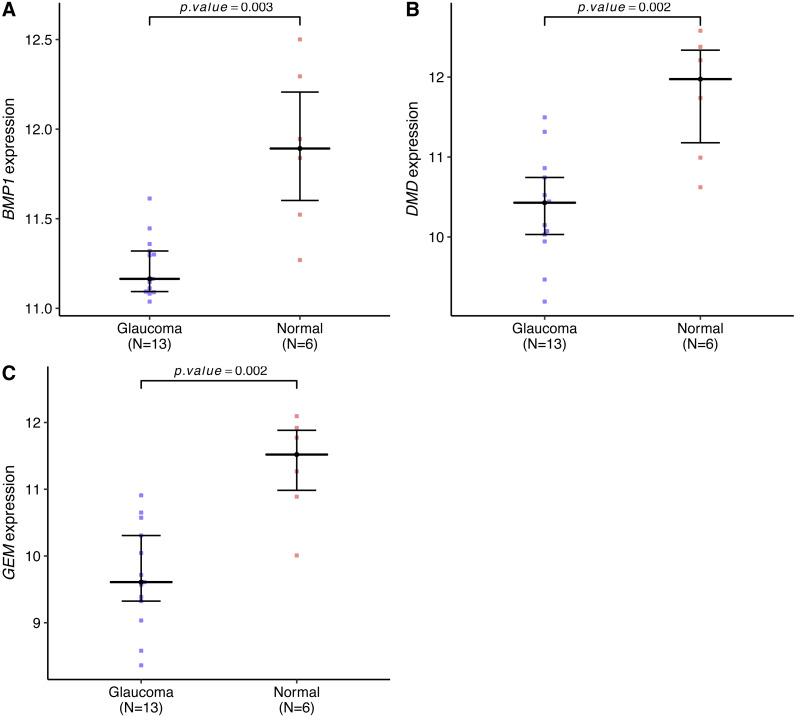
BMP1, DMD and GEM expression levels in the glaucoma group and normal group. The expression levels of all these key genes were significantly lower in the glaucoma group than in the normal group (A–C).

**Figure 8:**
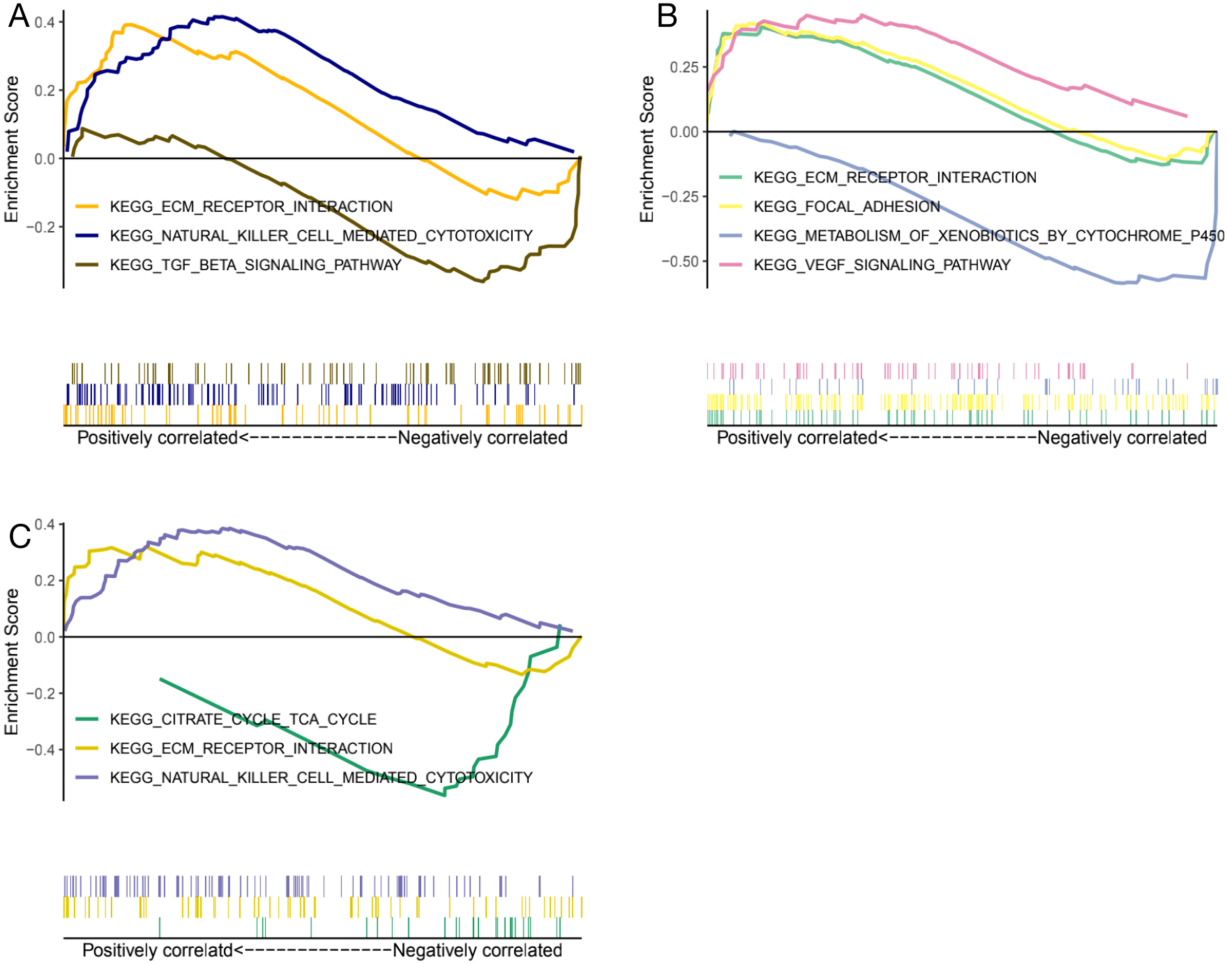
GESA enrichment of BMP1, DMD and GEM. BMP1 was significantly enriched in TGF-*β* signaling pathway, ECM receptor interaction, natural killer cell mediated cytotoxicity (A). DMD was significantly enriched in focal adhesion, ECM receptor interaction, VEGF signaling pathway (B). GEM was significantly enriched in ECM receptor interaction, natural killer cell mediated cytotoxicity (C).

Genes expression levels were shown in the Fig. 7. Interestingly, all these three genes were significantly expressed higher in the normal samples than in the glaucoma samples. Based on gene expression level, GESA was performed to identify the pathways associated with key genes. The enrichment scores of these differentially expressed genes in important terms were presented in Table 1. BMP1 was significantly enriched in TGF-β signaling pathway, ECM receptor interaction, natural killer cell mediated cytotoxicity (Fig. 8A). DMD was significantly enriched in focal adhesion, ECM receptor interaction, VEGF signaling pathway (Fig. 8B). GEM was significantly enriched in ECM receptor interaction, natural killer cell mediated cytotoxicity (Fig. 8C). Particularly worth noting is that all of the three key genes were enriched in the ECM receptor interaction, and both of *BMP1* and *GEM* were enriched in natural killer cell mediated cytotoxicity.

**Table 1:** Enrichment score s of differentially expressed genes in important terms.

GENE	NAME	ES	NES	NOM *p*-val
	KEGG_NATURAL_KILLER_CELL_MEDIATED_CYTOTOXICITY	0.41486168	1.4794924	0.01814516
BMP1	KEGG_HEMATOPOIETIC_CELL_LINEAGE	0.5492907	1.4255947	0.02554028
	KEGG_GLYCOSPHINGOLIPID_BIOSYNTHESIS_LACTO _AND_NEOLACTO_SERIES	0.58440936	1.4215839	0.03406814
	KEGG_ECM_RECEPTOR_INTERACTION	0.39169973	1.3057723	0.0492126
	KEGG_GLIOMA	0.47700384	1.8822955	0
	KEGG_RENAL_CELL_CARCINOMA	0.48509955	1.7634672	0.004
	KEGG_PANCREATIC_CANCER	0.46747884	1.6679943	0.00823045
	KEGG_FOCAL_ADHESION	0.42024386	1.6549476	0.00205339
	KEGG_NON_SMALL_CELL_LUNG_CANCER	0.39712295	1.6283306	0.02301255
	KEGG_HYPERTROPHIC_CARDIOMYOPATHY_HCM	0.57694465	1.6273366	0.00409836
	KEGG_VIBRIO_CHOLERAE_INFECTION	0.36380395	1.6116077	0.03846154
DMD	KEGG_DILATED_CARDIOMYOPATHY	0.49189743	1.5051197	0.02414487
	KEGG_ARRHYTHMOGENIC_RIGHT_VENTRICULAR _CARDIOMYOPATHY_ARVC	0.4962006	1.5024999	0.02857143
	KEGG_VEGF_SIGNALING_PATHWAY	0.45013994	1.480682	0.03617021
	KEGG_SMALL_CELL_LUNG_CANCER	0.3820947	1.4728578	0.02794411
	KEGG_MELANOMA	0.40423834	1.4425186	0.03607215
	KEGG_GAP_JUNCTION	0.3834123	1.4365592	0.0359408
	KEGG_PATHWAYS_IN_CANCER	0.31513023	1.3521765	0.036
	KEGG_ECM_RECEPTOR_INTERACTION	0.40342882	1.3490801	0.02663934
GEM	KEGG_GLYCOSPHINGOLIPID_BIOSYNTHESIS_LACTO _AND_NEOLACTO_SERIES	0.62023604	1.5112221	0.0155642
	KEGG_JAK_STAT_SIGNALING_PATHWAY	0.4429717	1.423994	0.0130841
	KEGG_AUTOIMMUNE_THYROID_DISEASE	0.6393696	1.4187429	0.02808989
	KEGG_PRIMARY_IMMUNODEFICIENCY	0.5353427	1.4012718	0.01801802

**Notes.**

ES enrichment score NES normalized enrichment score

### Validation of the key genes

**Figure 9:**
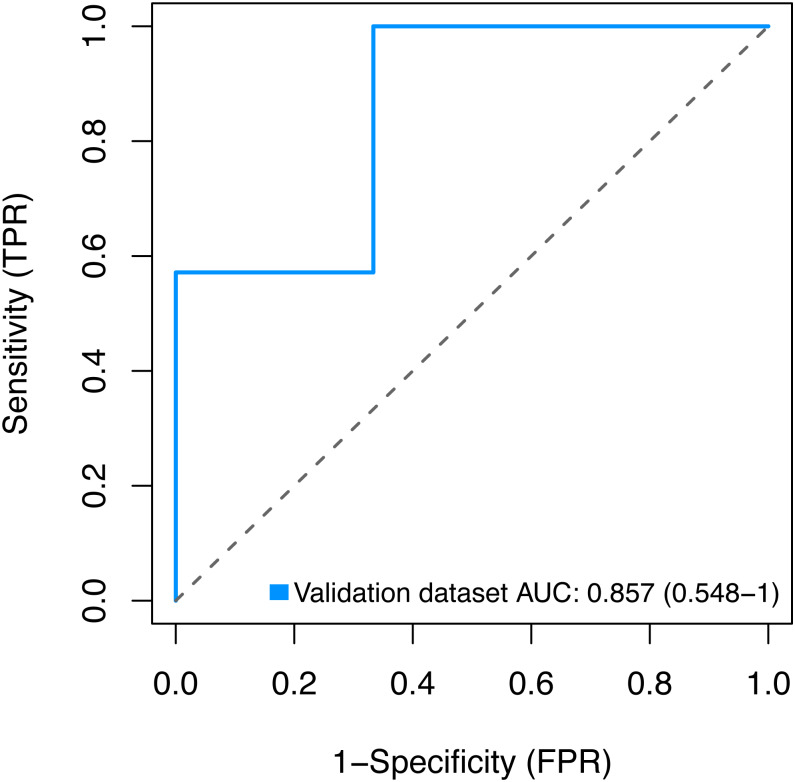
Validation for the key genes using the GSE2378 dataset. TPR, true positive rate, represent sensitivity. FPR, false positive rate, represent specificity.

Finally, we sought to validate the expression of the three key genes *BMP1*, *DMD* and *GEM* by using the GSE2378 dataset, and the high AUC value (85%) showed in Fig. 9 indicated the accurate discrimination of pathological and normal samples.

Our results indicate that *BMP1*, *DMD* and *GEM* are potential biomarkers of glaucoma. These genes were validated with a separate database. These findings may provide novel insight into the pathogenesis of glaucoma, and could be helpful for its early diagnosis, prevention, and treatment.

## Discussion

The occurrence and development of some types of glaucoma may be determined by several genes, especially in early-onset and adult-onset forms, such as developmental glaucoma, juvenile-onset primary open angle glaucoma, congenital glaucoma, and familial normal-tension glaucoma (Wang & Wiggs, 2014). Therefore, we tried to identify genes involved in glaucoma, instead of discuss existing therapies and known mechanisms. In this study, we used WGCNA to construct 10 coexpression modules containing 8,606 genes identified in 19 human optic nerve head samples in order to determine the relationships between the glaucoma transcriptome and clinic traits. As WGCNA is reportedly to be more reliable and more biologically significant than other methods (Chou et al., 2014), we used this method to form clusters of predictive functionally related genes. In this way, we identified modules and selected genes that might be considered as biomarkers for the diagnosis and/or treatment of glaucoma. Two coexpression modules were strongly associated with the clinical traits of glaucoma, particularly age. The green–yellow module was enriched in genes involved in cellular inflammation and injury, whereas the red module was mainly enriched in genes involved in tissue physiological processes and cell cycles.

After the two coexpression modules were constructed with WGCNA, we identified three key hub genes: *BMP1*, *DMD* and *GEM*. Based on genes expression presentation and results of GSEA analysis, we found that three key genes were significantly lower in glaucoma samples than in normal samples and they enriched in some of the same pathways. This further suggests that they may be potential biomarkers of the disease.

BMP1, also known as procollagen C-proteinase, is involved in the maturation of collagen, which is necessary for bone and cartilage growth and structural maintenance. It is synthesized throughout the human body, except in the brain (Brown & Goldstein, 1986). Loss-of-function mutations in *BMP1* result in abnormal collagen formation and occur in various autosomal recessive diseases associated with defective osteogenesis (Syx et al., 2015). BMP1 is also known to cleave extracellular macromolecules, including probiglycan (Scott et al., 2000) and prolaminin 5 (Amano et al., 2000), when the extracellular matrix is too excessive. Besides, BMP1 is necessary to generate functional high-density lipoprotein particles for reverse cholesterol transport (Riggs & Rohatgi, 2019), and contributes to renal fibrosis in chronic kidney disease by affecting the maturation and deposition of collagen and subsequent profibrotic responses and inflammation (Bai et al., 2019). Therefore, it is difficult to predict whether BMP1 has beneficial or deleterious effects in glaucoma.

*DMD*, with 79 exons and tightly regulated introns, is the largest protein-coding gene and encodes an important cytoskeletal protein. Mutations in *DMD* cause Duchenne muscular dystrophy (Hoffman, Brown Jr & Kunkel, 1987). DMD plays critical roles in the formation and maintenance of neuromuscular junctions (Ahn & Kunkel, 1993), and has been identified as a signaling protein involved in muscle contraction and other functions, as well as in dystrophin function (Anderson et al., 2002). It is prominently expressed in the eye and brain. Depending on the promoter used, *DMD* encodes both dystrophin and Dp71, which show different expression patterns during embryonic development in zebrafish, especially in the eye and germ-layer structures (Bolaños-Jiménez et al., 2001). Preliminary results have indicated that DP260 (and possibly DP71) is associated with abnormal b-wave amplitudes on dark-adapted electroretinography (ERG) in mice (Pillers et al., 1999). More-recent studies have demonstrated a strong association between *DMD* mutations that affect various DMD isoforms and abnormal scotopic ERGs and severe neurodevelopmental problems (Ricotti et al., 2016). Genetic studies have linked myocilin with open-angle glaucoma (Stone et al., 1997). Taken together, these findings provide strong evidence that *DMD* may be used as a biomarker of dystrophin function in glaucoma.

GEM is a small GTP-binding protein belonging to the RAS superfamily of monomeric G-proteins, with extensive biological functions. It is already widely accepted as a protein involved in rat obesity (Bourne, Sanders & McCormick, 1991). GEM has two key biological effects, depending on its site of expression: the inhibition of voltage-gated calcium channel activity and the inhibition of RHO-kinase-mediated cytoskeletal reorganization (Ward et al., 2004). Previous studies have shown that GEM is a regulatory protein that participates in receptor-mediated signal transduction at the plasma membrane (Maguire et al., 1994). It is widely expressed in many tissues and organs, including the spleen, lung and large intestine of the goat (Xu et al., 2018), and it is overexpressed in skeletal muscle in individuals with type 2 diabetes (Maguire et al., 1994). Here, we propose that the expression of *GEM* in the eye might also be clinically significant in glaucoma.

In conclusion, we have shown that the green–yellow module is the module most relevant to glaucoma. We also identified three key genes, *BMP1*, *DMD* and *GEM* that might be served as potential biomarkers of glaucoma. Although the biological and clinical relevance of these genes are not completely understood, they may be involved in a number of cellular processes relevant to the pathogenesis of glaucoma, such as inflammation or oxidative stress. Our results may also contribute to the development of novel approaches with great efficacy to diagnose and treat glaucoma, as they are genes of particular interest in relation to the development or progression of the disease.

There were certain limitations to our study, for example, the sample size was relatively small with Chinese ethnicity included only. Although we screened three key genes through a combination of multiple analyses, this study still lack experimental verification. To further unravel the function of these newly genes in the pathogenesis of glaucoma, studies with a larger sample size should focus on confirming the relevance between biological and clinical changes, and validating the value of these potential genetic targets by experiments to prevent the onset or the progression of glaucoma.

##  Supplemental Information

10.7717/peerj.9462/supp-1 Supplemental Information 1 The R language script for WGCNA analysis of all genes

10.7717/peerj.9462/supp-2 Supplemental Information 2 GSE2378 of normal and glaucomatous astrocytes

10.7717/peerj.9462/supp-3 Supplemental Information 3 GSE9944 of gene expression data on human optic nerve head astrocytes in Caucasian and African americans with or without glaucoma

## Additional Information and Declarations

### Data Availability

The following information was supplied regarding data availability: The data and code are available at GitHub: https://18211260008.github.io/glaucoma/. The data is also available at NCBI GEO: GSE9944, GSE2378.
